# Conifer Stored Resources and Resistance to a Fungus Associated with the Spruce Bark Beetle *Ips typographus*

**DOI:** 10.1371/journal.pone.0072405

**Published:** 2013-08-13

**Authors:** Eleanor C. Lahr, Paal Krokene

**Affiliations:** 1 Division of Biological Sciences, The University of Montana, Missoula, Montana, United States of America; 2 Norwegian Forest and Landscape Institute, Ås, Norway; Swedish University of Agricultural Sciences, Sweden

## Abstract

Bark beetles and associated fungi are among the greatest natural threats to conifers worldwide. Conifers have potent defenses, but resistance to beetles and fungal pathogens may be reduced if tree stored resources are consumed by fungi rather than used for tree defense. Here, we assessed the relationship between tree stored resources and resistance to *Ceratocystis polonica*, a phytopathogenic fungus vectored by the spruce bark beetle *Ips typographus.* We measured phloem and sapwood nitrogen, non-structural carbohydrates (NSC), and lipids before and after trees were attacked by *I. typographus* (vectoring *C. polonica*) or artificially inoculated with *C. polonica* alone. Tree resistance was assessed by measuring phloem lesions and the proportion of necrotic phloem around the tree's circumference following attack or inoculation. While initial resource concentrations were unrelated to tree resistance to *C. polonica*, over time, phloem NSC and sapwood lipids declined in the trees inoculated with *C. polonica*. Greater resource declines correlated with less resistant trees (trees with larger lesions or more necrotic phloem), suggesting that resource depletion may be caused by fungal consumption rather than tree resistance. *Ips typographus* may then benefit indirectly from reduced tree defenses caused by fungal resource uptake. Our research on tree stored resources represents a novel way of understanding bark beetle-fungal-conifer interactions.

## Introduction

Symbiotic relationships between blue-stain fungi and bark beetles have fascinated researchers since they were first described more than 100 years ago [1]–[5], and conifer resistance to beetles and fungi has been the subject of much research [6]–[12]. Still, the adaptive basis for the relationship between bark beetles and blue-stain fungi has not been fully determined. One basis for bark beetle-fungal mutualisms may be that fungal phytopathogenicity helps beetles to overcome conifer defenses [13], [14], while alternatively, some mutualistic fungi provide important nutritional benefits to their beetle partner and do not seem to engage tree defenses [5], [15]–[19]. These different perspectives on the nature of beetle-fungal mutualisms may in fact be complementary because ultimately, bark beetles and their different fungal associates must all overcome tree resistance and obtain nutrients from the tree in order to thrive. Regardless of the proximate and ultimate basis for bark beetle-fungal mutualisms, mobilization of tree resources is a prerequisite for successful tree resistance and also for successful colonization of the tree by bark beetles and fungi.

While there have been studies on environmental factors that influence colonization of conifers by blue-stain fungi, such as temperature, oxygen level, and tree water potential [20]–[23], less is known about how tree stored resources influence fungal colonization or tree resistance. Conifers coordinate multiple defenses to resist bark beetles and blue-stain fungi, including constitutive defenses, which are established before bark beetle attack and represent a fixed cost, and inducible defenses, which are mobilized in response to a beetle attack [13], [24]. Conifer defensive chemicals are primarily carbon based, including phenolics and terpenoid compounds [13], [25], [26]. Stored carbon reserves of non-structural carbohydrates and lipids may contribute to the activation of induced defenses like traumatic resin ducts and polyphenolic parenchyma cells, which are important components of resistance in Norway spruce and other conifers, while stored nitrogen may be an important contributor to the enzymatic and biosynthetic pathways underlying conifer defense [25]–[29]. However, stored mobile carbon compounds and nitrogen compounds also provide a potential food source for invading fungi [30]–[32], and conifer resistance to fungal pathogens may thus be complicated by the potential for tree stored resources to enhance fungal performance as well as tree resistance. Specifically, mobilization of stored resources for inducible defenses that provide long-term resistance could be precluded by fungal consumption of tree resources or by fungal redistribution of stored resources to benefit developing beetle larvae. A better appreciation of how tree resources may benefit the fungi is necessary to understand bark beetle-fungal interactions and their consequences for conifer resistance.

Here, we evaluate the relationship between conifer stored resources and resistance to the virulent fungal pathogen *Ceratocystis polonica* vectored by the spruce bark beetle *Ips typographus*. This beetle is one of the most aggressive and destructive forest insect pests in Northern Europe and has killed millions of trees in periodic outbreaks [33]–[35]. Its main host tree, Norway spruce (*Picea abies*), is ecologically and economically important across Europe, and the pathogenicity of *C. polonica* to Norway spruce is well documented [10], [11], [27], [36]–[38]. In southern Norway, where this study took place, *C. polonica* is one of the most frequent fungal associates of *I. typographus* [33], [34], [39], [40]. We use the term “resistance” to refer to the overall ability of a tree to overcome the challenge of *C. polonica* colonization, an ability that can vary with tree age and factors associated with age, such as size, architecture, and functional priorities [41]. The size of phloem lesions and the proportion of necrotic phloem around the tree's circumference following fungal infection are measures of tree resistance and represent the tree's attempt to compartmentalize and kill a fungal pathogen via the hypersensitive response [6], [13].

**Figure 1:**
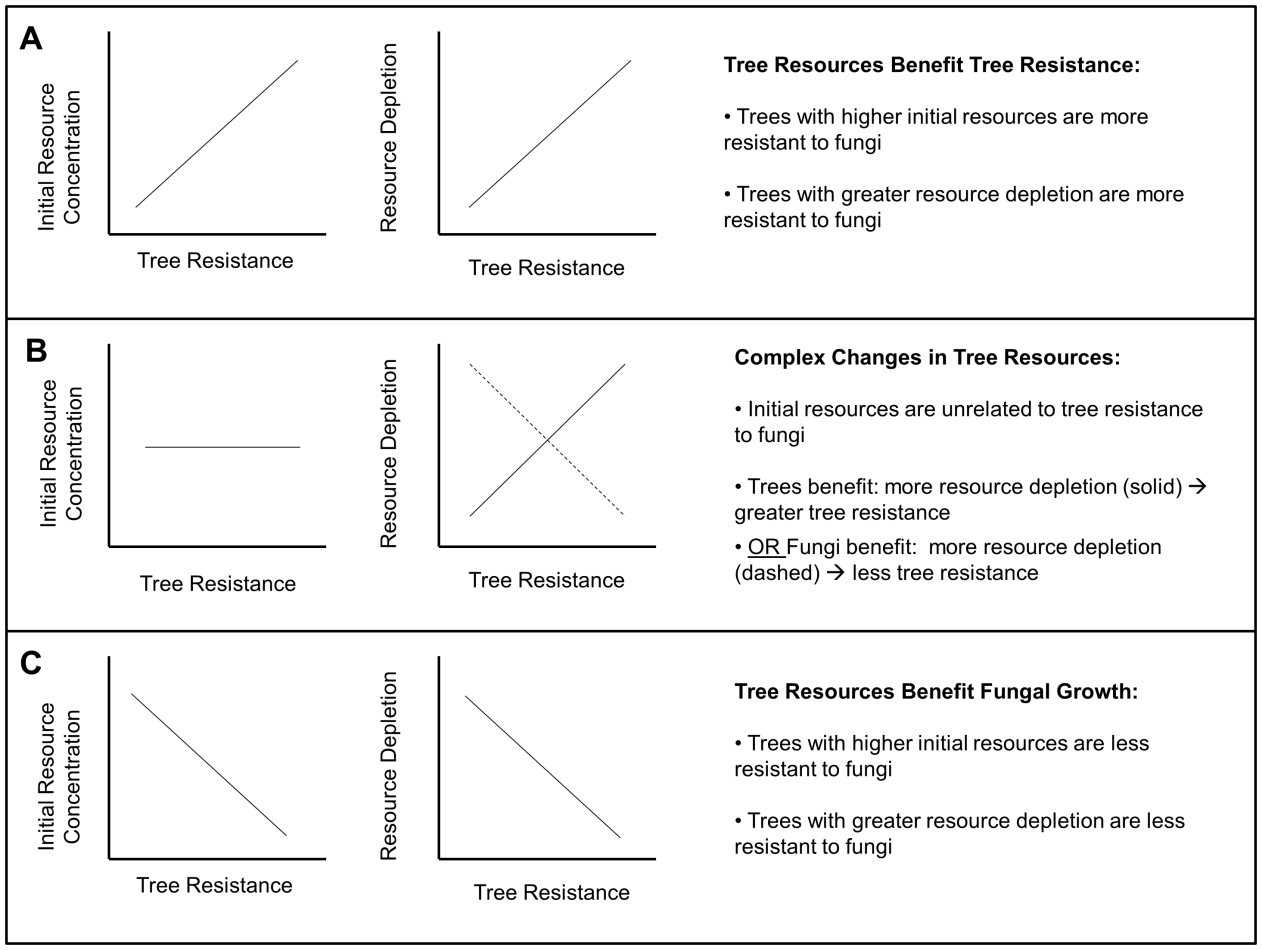
Predictions illustrating possible changes in tree stored resources in relation to tree resistance. (A) If stored resources are more beneficial to the tree than the fungus, trees with higher initial resource concentrations and more resource depletion over time will be more resistant to fungal colonization. (B) Tree resistance may be independent of initial resource concentrations and cause complex changes in tree resources over time. Independent of initial resource concentrations, more resistant trees may have more resource depletion and successfully resist fungal colonization (solid line). Alternatively, less resistant trees may have more resource depletion because fungi consume resources (dashed line). (C) If stored resources are overall more beneficial to the fungus than to the tree, trees with higher initial resource concentrations and more resource depletion over time will be less resistant to fungal colonization.

To determine the relationship between tree stored resources and tree resistance or fungal consumption of resources, we developed the following predictions: (1) If stored resources benefit tree resistance more than they benefit *C. polonica* as a food source, trees with more initial resources and larger resource depletion over time will be more resistant to fungal colonization (Figure 1A). (2) If stored resources do not have a net influence on tree resistance, resistance will be independent of initial resource concentrations and resource depletion may benefit trees or fungi (Figure 1B). (3) If stored resources benefit *C. polonica* more than the tree, trees with higher initial resource concentrations and larger resource depletion over time will be less resistant to fungal colonization (Figure 1C). To test these predictions we measured nitrogen and two mobile carbon compounds (non-structural carbohydrates (NSC) and lipids) before and twice after Norway spruce trees were attacked by *I. typographus* (vectoring *C. polonica*) or artificially inoculated with *C. polonica.* Our control, beetle attack, and fungal inoculation treatments were applied to genetically identical Norway spruce clones, allowing us to compare resource concentrations over time with measures of tree resistance, without the added variability between treatments caused by tree age or genetic background.

## Materials and Methods

### Study site and treatments

This study was conducted at Hogsmark Experimental Farm, Ås, Norway, a research forest owned and operated by the Norwegian Forest and Landscape Institute. Permission for this research was granted by the Norwegian Forest and Landscape Institute. Three ramets each from nine Norway spruce clones were selected and allocated to the following treatments: control, attack by *Ips typographus*, or inoculation with *Ceratocystis polonica* (Table 1). Trees were 51 years old at the time of the experiment. Mean tree diameter at breast height (1.4 m above the ground) was 20.6±3.3 cm (mean ± standard deviation) and did not differ significantly between treatments (F_(2, 24)_ = 1.108, p = 0.346). Mean daily temperatures at the study site ranged from 10.4–17.4°C in June, 5.8–13.2°C in August, and 0–6.2°C in November.

**Table 1:** Treatment details and outcome for individual Norway spruce trees attacked by *Ips typographus*, inoculated with the fungus *Ceratocystis polonica*, or left untreated as control.

Treatment	Clone	Diameter (cm)	Inoculations (number)	Necrotic Phloem (%)^a^	Phloem Lesions (cm)^b^	Outcome^c^
Attack	108	26.7	–	35	–	alive
Attack	109	24.0	–	11	–	alive
Attack	113	21.0	–	20	–	alive
Attack	114	27.2	–	32	–	alive
Attack	123	21.2	–	12	–	alive
Attack	124	16.7	–	100	–	dead
Attack	125	17.8	–	31	–	alive
Attack	127	22.3	–	53	–	alive
Attack	129	19.6	–	100	–	dead
Inoculation	108	19.4	293	100	4.5±0.7	dead
Inoculation	109	23.6	355	50	5.0±2.1	alive
Inoculation	113	19.6	295	98	8.0±1.5	dying
Inoculation	114	22.6	341	95	7.9±2.5	dying
Inoculation	123	21.2	319	45	6.5±1.1	alive
Inoculation	124	17.2	259	100	8.3±0.9	dead
Inoculation	125	16.7	252	95	7.0±0.9	dying
Inoculation	127	20.2	305	100	9.1±1.6	dead
Inoculation	129	15.6	235	100	8.8±1.5	dead
Control	108	24.5	–	0	–	alive
Control	109	23.1	–	0	–	alive
Control	113	23.2	–	0	–	alive
Control	114	22.6	–	0	–	alive
Control	123	21.3	–	0	–	alive
Control	124	16.2	–	0	–	alive
Control	125	17.4	–	0	–	alive
Control	127	20.1	–	0	–	alive
Control	129	15.1	–	0	–	alive

a Control trees were not measured, but healthy trees generally have no necrotic phloem (e.g. Krokene and Solheim 1998).

b Phloem lesion length is mean ± standard deviation.

c Outcome is a qualitative assessment of tree health one year after the start of the experiment.

The nine trees in the “beetle attack” treatment were baited with attractant pheromone on May 30, 2010 (Ipslure, Borregaard Inc., Sarpsborg, Norway), and were attacked by *I. typographus* the following week. In this part of its range *I. typographus* is univoltine, with tree colonization, reproduction, and larval development occurring during the summer, from late May to August. New adults leave the tree in autumn, overwinter in the ground, and re-emerge and attack new trees the following spring when maximum daytime temperatures reach 19–20°C. Beetle attack densities on trees were not measured, but seven trees in the beetle attack treatment had low attack densities and beetle colonization was unsuccessful. Two trees were attacked at densities typically occurring during an *I. typographus* attack (400 attacks m^−2^; [36], [42]) and these two trees were successfully colonized. The nine trees in the “inoculation” treatment were inoculated with *C. polonica* (isolate NFLI 93–208/115) on June 9–10, 2010. Trees were mass-inoculated at a density of 400 inoculations m^−2^ around the main bole, from 0.8–2.0 m above the ground. Inoculations were performed by removing a bark plug with a 5 mm cork borer, inserting inoculum, and replacing the bark plug. Inoculum consisted of actively growing mycelium of *C. polonica* cultured on malt agar (2% malt and 1.5% agar). The number of fungal inoculations made per tree is shown in Table 1. Trees in the control treatment were not manipulated.

The resistance of inoculated trees was evaluated by measuring the length of five phloem lesions at the top and at the bottom of the inoculation band on November 1, 2010 (Table 1). Smaller phloem lesions indicate greater resistance to fungal pathogens [42]. Phloem lesions were too small or inconsistently occurring for measurement in the trees where beetle colonization was unsuccessful, described above, so to obtain comparable data on tree resistance across treatments we carefully shaved off the cork bark and measured the percentage necrotic phloem around the stem circumference at breast height of all 18 attacked and inoculated trees on June 9, 2011 (Table 1). Control trees were not measured, but since the trees were healthy-looking with no previous wounds or scars they were assumed to have no necrotic phloem [42].

### Sample collection and biochemical analysis

Phloem and sapwood were collected from all trees at three time points; (1) on May 30, 2010, prior to any treatment; (2) on August 18, 2010, shortly after the next *I. typographus* generation was expected to be fully developed; and (3) on October 16, 2010, near the end of the growing season. At each time point we collected one phloem sample using a 30 mm diameter arch punch, and 2–3 samples of the entire sapwood depth using a 5 mm hand increment borer, from each tree at breast height. Samples were oven dried at 75°C for 48 hours, and ground to powder in an IKA A11B grinder followed by an IKA MF10 grinder with a 0.5 mm mesh screen (IKA, Staufen, Germany).

We measured sapwood lipids (acylglycerols) and phloem and sapwood nitrogen and non-structural carbohydrates (NSC; glucose, fructose, sucrose, and starch). Lipid analysis was performed only for sapwood tissue, as lipid translocation and storage is much less significant in phloem tissue [43]. Lipid concentrations were analyzed using a photometric method [44]. Briefly, 10–14 mg wood powder was extracted in 1 mL aqueous NaOH for 30 minutes, and glycerol was converted to glycerol-3-phosphate. The amount of liberated glycerol was determined in a 96-well microplate reader at 340 nm (model EL800, BioTek Instruments Inc, Winooski, Vermont, U.S.A.). NSC in the phloem and sapwood was analyzed using a similar photometric method [45]. Briefly, 12–14 mg of wood or bark powder was extracted in 1.6 mL distilled water at 100°C for one hour. An aliquot of this extract was used to determine low molecular weight carbohydrates following enzymatic breakdown of fructose and sucrose to glucose. Enzymatic breakdown of starch to glucose by a fungal amylase (‘Clarase’, Genencor International Inc., Rochester, New York, U.S.A.) was done using a second aliquot of wood or bark extract. This enzymatic digest occurred at 40°C overnight. Glucose was converted to gluconate-6-phosphate, and this conversion was measured in a 96-well microplate reader at 340 nm. Sample nitrogen content was measured using an elemental analyzer (model EA 1110, CE Instruments, Wigan, U.K.) at the University of Montana Environmental Biogeochemistry Laboratory.

### Statistical analysis

**Figure 2:**
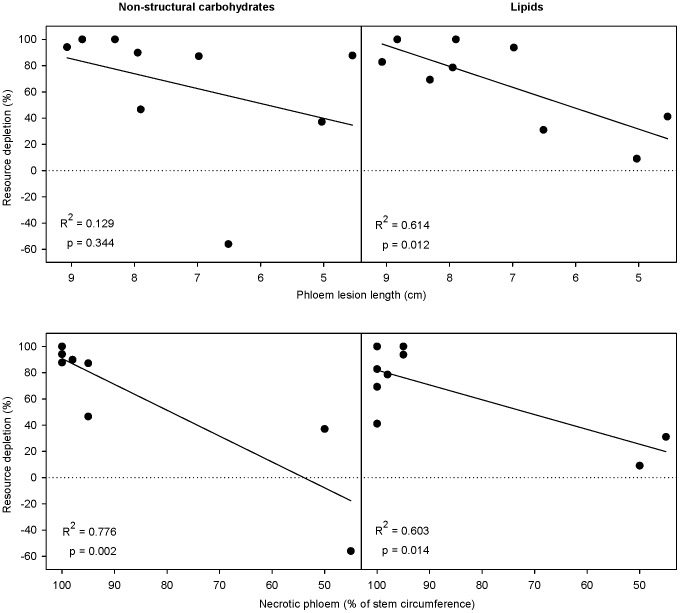
Correlations between tree resource change and resistance following inoculation of the fungus *Ceratocystis polonica*. Percentage change in phloem non-structural carbohydrates (left panels) and sapwood lipids (right panels) from May to October is shown. Norway spruce resistance was measured as phloem lesion length (upper panels) and percentage necrotic phloem around the stem circumference (lower panels). Shorter lesions and less necrotic phloem signify a more resistant tree. Dotted lines show equal resource levels in May and October, whereas positive values denote a decline in resource levels over time (i.e. resource depletion). Linear trend lines are shown for clarity.

**Figure 3:**
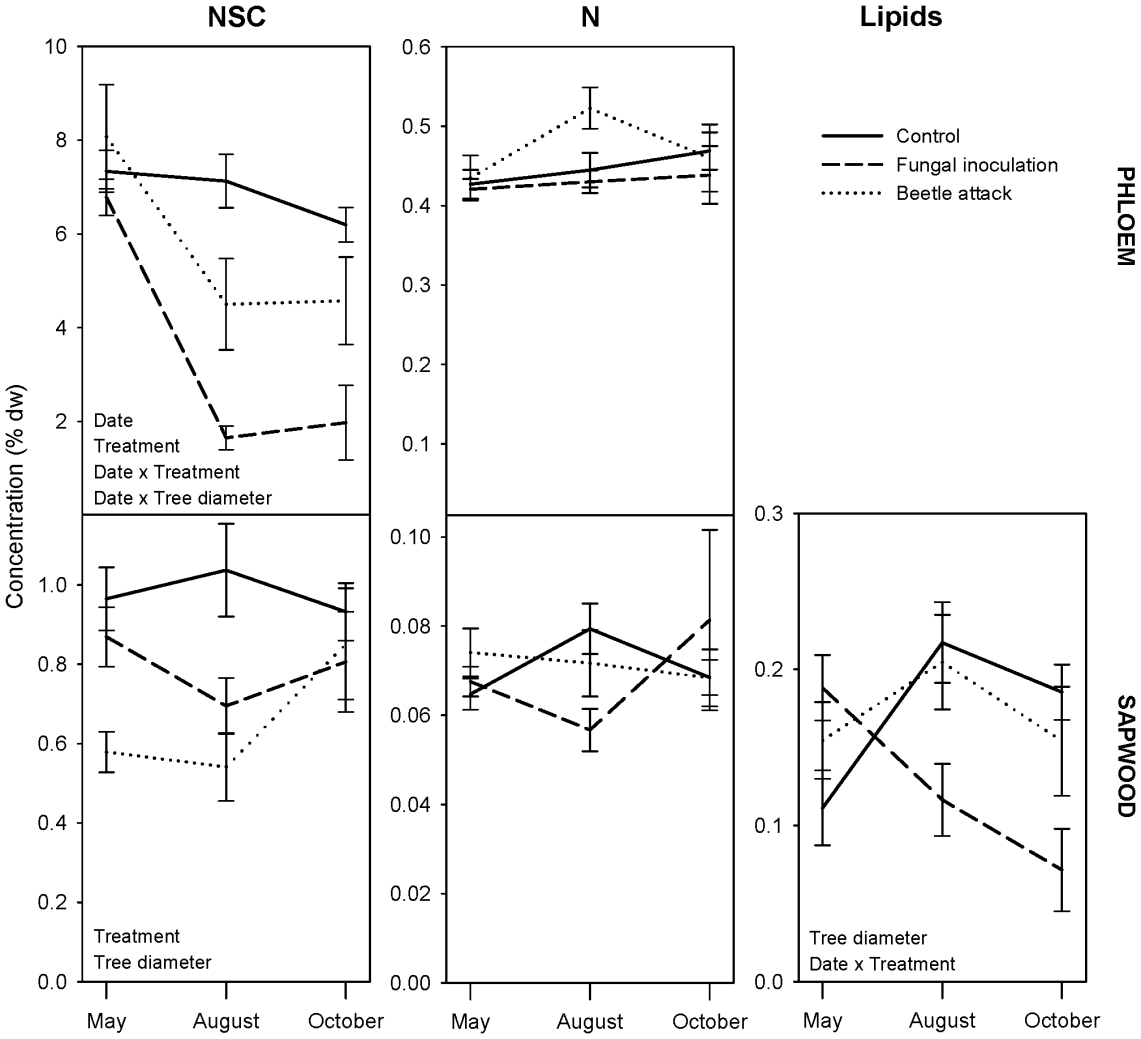
Change in tree resources following treatment. Change in non-structural carbohydrates (NSC) and nitrogen (N) in the phloem and sapwood and change in lipids in the sapwood is shown for control, fungal-inoculated, and beetle-attacked trees. Error bars indicate ±1 standard error. Significant ANOVA effects are indicated in the lower left corner of the panels. Note differences in y-axis scale between the sapwood and phloem for NSC and nitrogen.

Bivariate correlations were used to compare phloem lesion length and the percentage of necrotic phloem with initial tree resource concentrations and with the percentage change in resource concentrations over time (Figure 2). A general linear model with repeated measures was used to evaluate the effect of treatment on tree resource concentrations over time (Table 2, Figure 3). Sampling date was a repeated measures factor and tree diameter was included as a covariate. Response variables included sapwood lipids, nitrogen, and NSC, and phloem nitrogen and NSC (Table 2). Due to a lack of correlation among response variables (for eight of nine correlations, |r|≤0.161), multiple univariate tests were performed instead of a multivariate approach. Statistical analyses were performed in PASW Statistics 18 (IBM Statistics).

**Table 2:** General linear model with repeated measures showing effects on resource concentrations in Norway spruce sapwood and phloem.

		Sapwood			Sapwood			Sapwood			Phloem			Phloem	
		Lipids			NSC			Nitrogen			NSC			Nitrogen	
	df	F	p	df	F	p	df	F	p	df	F	p	df	F	p
Intercept		0.105	0.749		4.042	0.056		8.467	0.009		6.055	0.022		41.118	0.000
Treatment	2, 23	0.888	0.425	2, 23	12.172	**0.000**	2, 23	0.007	0.993	2, 23	10.272	**0.001**	2, 23	1.379	0.276
Diameter	1, 23	5.359	**0.030**	1, 23	4.796	**0.039**	1, 23	0.102	0.753	1, 23	0.013	0.909	1, 23	0.336	0.569
Date	2, 22	0.579	0.454	2, 22	0.244	0.786	2, 18	1.439	0.263	2, 22	8.538	**0.002**	2, 18	2.317	0.144
Date × Treatment	2, 22	9.770	**0.001**	2, 22	1.411	0.236	2, 18	2.314	0.095	2, 22	3.328	**0.018**	2, 18	1.526	0.243
Date × Diameter	4, 44	0.617	0.440	4, 44	0.366	0.697	4, 36	1.424	0.267	4, 44	5.396	**0.012**	4, 36	1.920	0.182

Treatment (control, beetle attack, fungal inoculation) is a factor, sampling date is a repeated measures factor, and tree diameter is a covariate. Degrees of freedom (df) are numerator, denominator. Bold p-values indicate significant effects.

## Results

The proportion of necrotic phloem around the tree's circumference and the length of phloem lesions varied between Norway spruce clones in the fungal inoculation treatment, indicating variation between clones in overall resistance to *C. polonica* (Table 1). The percentage of necrotic phloem around the tree's circumference was generally high in the fungal inoculation treatment; seven of nine trees had more than 95% necrotic phloem, and these seven trees appeared to be dead or dying one year following treatment (Table 1). In contrast, only three of nine trees attacked by *I. typographus* had more than 50% necrotic phloem, and only two of those trees appeared dead by the following year (Table 1).

Both measures of tree resistance to *C. polonica* inoculation, the proportion of necrotic phloem around the tree's circumference and phloem lesion lengths, were negatively correlated with percentage resource change following inoculation (Figure 2). The trees with highest percentage of necrotic phloem (trees with less resistance) had the greatest depletion of phloem NSC and sapwood lipids from May to October (Figure 2). Similarly, trees with the largest phloem lesions had the greatest decline in sapwood lipids from May to October (Figure 2). Change in phloem NSC from May to October was not correlated with phloem lesion length (Figure 2), but trees with larger phloem lesions had a near-significant correlation with a decline in phloem NSC from May to August (R^2^ = 0.399, p = 0.068). Other significant correlations showed that trees with more necrotic phloem had a greater decline in phloem NSC from May to August (R^2^ = 0.613, p = 0.013) and trees with larger phloem lesions had a greater decline in sapwood nitrogen from May to August (R^2^ = 0.444, p = 0.050). Overall, tree resistance to *C. polonica* was not significantly correlated with initial resource concentrations (R^2^ = 0.000 - 0.272, p = 0.150 - 0.979, for phloem lesion length; R^2^ = 0.012 - 0.348, p = 0.094 - 0.783, for percentage necrotic phloem).

Treatment (beetle attack, fungal inoculation, control) had some independent effects on tree resource concentrations, and also interacted with sampling date to influence resource concentrations over time (Table 2, Figure 3). A pronounced decline in phloem NSC occurred following beetle attack and, in particular, fungal inoculation. This decline occurred entirely between May and August in both treatments. Sapwood NSC concentrations were more variable; beetle-attacked trees significantly increased in NSC, but NSC was also initially lower in this treatment. A significant treatment × date interaction occurred for sapwood lipids, which declined consistently from May to October in fungal-inoculated trees. No significant changes in nitrogen occurred in any treatment.

## Discussion

While conifer defense against bark beetles and pathogens has been the subject of much research [6], [13], [14], [46], the potential for tree stored resources to enhance fungal performance rather than tree resistance has received little attention. In this study, we assessed three resource-change scenarios (Figure 1) that could occur following the introduction of the beetle-vectored fungus *C. polonica* to Norway spruce. We found that although initial tree resource concentrations were unrelated to tree resistance to *C. polonica*, over time, phloem non-structural carbohydrates (NSC) and sapwood lipids declined in the trees inoculated with *C. polonica* (Figure 3). Furthermore, trees that were less resistant (those with larger phloem lesions or more necrotic phloem) had greater resource declines (Figure 2). Overall, these data on resource depletion correspond with the scenario described by Figure 1B and suggest that at an inoculation density that leads to successful colonization, tree stored resources were consumed by fungi instead of being mobilized for tree resistance via carbon-based inducible defenses.

Interestingly, tree resistance to *C. polonica* was unrelated to initial resource concentrations, even though conifers have effective constitutive defenses against fungal colonization that include physical and chemical barriers [13], [29], [37], [47] and much research has linked bark beetle host preference with diminished tree defenses [7], [48]. Our Figure 1B describes two different scenarios that may result from this starting point. Independent of initial resource concentrations, more resource depletion over time may occur because trees mobilize their resources and resist fungal colonization, or more resource depletion over time may occur because fungi consume more resources, leaving trees less resistant. Our data support the latter scenario, which could occur if resource consumption enhances fungal colonization to such an extent that tree resistance breaks down, or if fungal consumption of resources prevents tree resistance. Overall, stored resource appear more beneficial to *C. polonica* than to the host tree.

We also observed greater declines in phloem NSC and sapwood lipids in trees inoculated with *C. polonica* than in trees attacked by *I. typographus* vectoring *C. polonica* (Figure 3). These differences between treatments may have occurred because fungal growth in beetle-attacked trees was initially inhibited by constitutive tree defenses against *I. typographus*, or because *I. typographus* may introduce other, less pathogenic fungi in addition to *C. polonica* [40] that could reduce the abundance of *C. polonica*. However, *I. typographus* predominantly vectors *C. polonica* in this area of southern Norway [39], [40]. Instead, treatment differences were likely caused by weak beetle attacks on the experimental trees due to above average temperatures in May 2010 that stimulated early beetle emergence. Many beetles had likely already emerged from hibernation when we placed our pheromone lures, resulting in lower than expected attack density on our experimental trees.

Successful beetle and fungal colonization occurred in two of nine beetle-attacked trees, and similar to the fungal inoculation treatment, these two trees had a high percentage of necrotic phloem and appeared dead by the following year (Table 1). Also similar were the large declines in phloem NSC and sapwood lipids that occurred in the two successfully attacked trees; however the small sample size prevents statistical comparison. Treatment differences might also occur if fungal performance improves in the absence of beetles, provided that fungal inoculation density is above the threshold required for successfully overcoming tree resistance [36]. Improved fungal performance in the absence of beetles could occur if there is competition for resources between these organisms at any time during their close association in the host tree. For example, even bark beetles and fungi with a mutualistic relationship overall [16], [19] may potentially compete for food resources within the tree if beetles ingest phloem tissue before it is colonized by fungi.

When *I. typographus* vectors *C. polonica*, the effects of fungal resource consumption are unlikely to provide direct nutritional benefits to developing beetle larvae. Although we cannot rule out the possibility that *C. polonica* or other associated fungi provide nutritional benefits to *I. typographus*, we did not observe an increase in phloem resource concentrations following fungal inoculation that would support this. Specifically, we observed no increase in nitrogen in phloem colonized by fungi (Figure 3). Nitrogen is an important limiting nutrient in insect development [49], [50] and fungi associated with other bark beetles have been shown to increase the nitrogen concentration in the phloem around developing beetle larvae [16], [19]. But, unlike bark beetles that do benefit from fungal-derived nutrients, *I. typographus* has a shorter development time in the tree (typically 2–3 months) and overwinters in the ground rather than the host tree. These differences could inherently limit the ability of *I. typographus* larvae to benefit from *C. polonica* within the time period that the two organisms share the host tree. Particulars of the bark beetle life cycle may therefore be important to consider when generalizing about nutritional benefits versus pathogenicity in bark beetle-associated fungi. However, even without providing direct nutritional benefits, resource uptake by fungi may indirectly benefit *I. typographus* by precluding tree allocation to resistance, by enhancing fungal growth to such an extent that tree defenses are overwhelmed [14], or by affecting tree function via interruption of water transport [51]–[54]. These possible effects of fungi on the host tree could all indirectly benefit *I. typographus* and deserve further study.

Conifer resistance to bark beetles and fungi is the subject of a great deal of research and this study demonstrates that insights into tree resource dynamics may improve our understanding of conifer resistance to fungal pathogens. While we observed changes in tree stored resource concentrations following fungal inoculation (Table 2, Figures 2 & 3), our results do not suggest that stored resources benefit host tree resistance (Figure 1A). Instead, our data suggest that tree stored resources may benefit fungi (Figure 1B). This in turn could indirectly benefit beetles by reducing tree resource allocation to defense or via negative effects of fungal growth on tree function. Additional research is needed to further investigate these effects in other bark beetle systems, and the predictions outlined in Figure 1 may prove useful in understanding tree resistance to bark beetles and fungi in other conifer species.
